# Oral Bioaccessibility and Exposure Risk of Metal(loid)s in Local Residents Near a Mining-Impacted Area, Hunan, China

**DOI:** 10.3390/ijerph15081573

**Published:** 2018-07-25

**Authors:** Ping Zhuang, Shuo Sun, Yingwen Li, Feng Li, Bi Zou, Yongxing Li, Hui Mo, Zhian Li

**Affiliations:** 1 Key Laboratory of Vegetation Restoration and Management of Degraded Ecosystems, South China Botanical Garden, Chinese Academy of Sciences, Guangzhou 510650, China; sunshuo16@scbg.ac.cn (S.S.); liyw@scbg.ac.cn (Y.L.); lifeng214@scbg.ac.cn (F.L.); Zoubi@scbg.ac.cn (B.Z.); liyongxing.7@163.com (Y.L.); mohui@scib.ac.cn (H.M.); 2 University of Chinese Academy of Sciences, Beijing 100049, China

**Keywords:** bioaccessibility, cadmium, arsenic, lead, exposure risk, rice

## Abstract

Metal(loid) contamination of food crops and soils resulting from mining activities has been a major concern due to the potential risk to humans. In this study, a total of 36 rice (home-grown and market rice), 38 vegetable, 10 drinking water, 4 river water, 18 soils and 30 urine samples were collected from an abandoned mining area or the local residents in China. Results showed that metal(loid) levels in some of the soil and drinking water samples exceeded the Chinese standard. Rice Cd concentration, rice Pb levels, and vegetable Pb levels exceeded the maximum permissible concentrations in 49%, 68%, and 42% of the samples, respectively. In gastric phases, the average Cd, Pb and As bioaccessibilities in rice were 72%, 70%, and 82%. In gastrointestinal phases, the average Cd, Pb and As bioaccessibilities in rice were 49%, 39%, and 94%. Vegetables (pak choi was selected) showed lower metal(loid) bioaccessibility than rice. The median concentrations of Cd, Pb and As in urine were 3.99, 4.82 and 64.8 µg L^−1^, respectivley. Rice had the highest contribution rates of Cd and Pb for daily intake, accounting for 114% and 210%, respectively. Vegetables contributed less, and very little contribution came from drinking water. Based on the bioaccessibility data, metal(loid) contamination around the mining area poses a great exposure risk to the local residents through consumption of food crops.

## 1. Introduction

With rapid industrialization and urbanization, soil contamination with metal(loid)s has become a great concern in China. A national survey in 2014 showed that 19.4% of arable land soils was contaminated, with metal(loid)s as the major pollutants [1]. Specifically, metal(loid) contamination in agricultural soils close to both active and abandoned mining sites is recognized as a serious problem [2]. Chronic exposure to metal(loid)s can have adverse effects on humans [3]. Studies show that Cd, Pb, and As are non-essential elements, likely causing mutagenic, teratogenic and carcinogenic effects at low exposure levels [4,5]. For example, in the Van region of Turkey, the high prevalence of upper gastrointestinal cancer rates is associated with high concentrations of toxic metals in the soils and vegetables [6]. In general, there are multiple exposure pathways throughout the world to harm human health, including direct oral ingestion of soils [7], water [8], rice [2], vegetables [9], and even dust [10] from mining areas.

In South China, due to the elevated inputs of metal(loid)s caused by mining activities, exceedance of the metal(loid) limits in food crops is widespread and serious [1]. A recent market basket survey in 2014 by Yuan et al. [11] showed that most of the food samples collected from 31 provinces of China have high Cd levels, and Zhu et al. [12] reported that Cd concentration in 65% of rice samples collected from rural areas of South China exceeded the maximum permissible value. Zhuang et al. [2] demonstrated that elevated Pb and Cd concentrations in leafy vegetables and rice samples from rural areas around mining sites exceeded the maximum permissible levels. It has been recognized that dietary intake is the main pathway of metal(loid)s exposure for most populations [13]. Thus, particular attention should be focused on the potential environmental hazards around mining-impacted areas.

Monitoring biological exposure, metal(loid)s or U-metal(loid)s, have been frequently used both in occupational investigation, as well as in human health [14]. Urinary metal(loid)s excretion is a biomarker of dietary metal(loid)s exposure and a significant increase in urinary metal(loid)s levels has been reported for adults, pregnant women, and children after food crops consumption [15]. It has been documented that nonrenal effects, such as increased risk of hypertension, cancer and cardiovascular diseases, poor neurodevelopment, and osteoporosis, were related to elevated urinary Cd [16]. In addition to measurement of urinary metal(loid)s, estimation of dietary metal(loid) intake provides information to reflect metal(loid)s body burden and potential human effects. Dietary metal(loid) intake is generally evaluated using total metal(loid) concentrations in foods and the corresponding consumption rate without taking account of metal(loid)s’ bioavailability or bioaccessibility (i.e., the fraction of metal(loid)s in food released from the food matrix or absorption in the systemic circulation) [17]. Currently, there is a lack of information on the bioaccessibility of metal(loid)s in the food matrix from mining areas based on in vitro digestion [18,19]. 

The main aims of the present study are: (1) to measure the concentrations of Cd, Pb and As in different environmental and urine samples, including rice, vegetable, drinking water, river water, soil samples collected from local farmers, etc.; (2) to study the Cd, Pb and As bioaccessibility in rice and vegetables grown in the contaminated sites; and (3) to determine human exposure risks based on the bioaccessible metal(loid)s of food crops and water via the ingestion pathway.

## 2. Materials and Methods

### 2.1. Study Area

**Figure 1:**
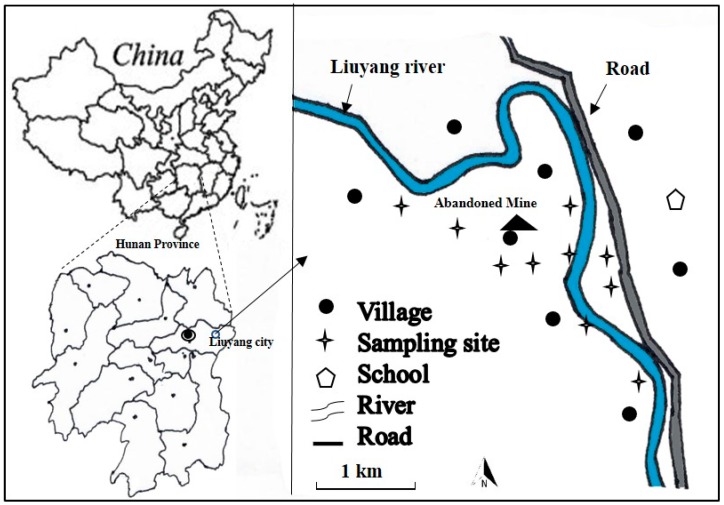
Locations of the study site.

The study area is located in the vicinity of an abandoned mining area (28°11′58″ N, 113°47′12″ E) in Liuyang City, Hunan, South China (Figure 1). The metal mine was privately exploited about 70 years ago. This area has a humid subtropical climate with an annual average temperature of 15 °C and annual rain fall of 1400 mm. Due to mining activities, the local farmland was polluted by the irrigation of discharged wastewater and the accumulation of wastes. Liuyang City is considered a livestock and poultry pollution area, where irrigation water contains high levels of contaminants from domestic animals’ feces. Local farmers live and grow rice and vegetables in farmlands surrounded by the abandoned mines. The home-grown rice and vegetables are predominantly consumed by local residents. Thus, there is a high potential of both elevated metal(loid)s concentration in food crops, as well as exposure risk to the farmers.

A questionnaire was distributed in 2016 to randomly selected participants (*n* = 30) living in three villages of the area around the abandoned mine to collect information on the amount of daily rice and vegetable consumption, type of the rice and vegetables grown at farm, and demographics, including gender, body weight, age (26–80 years old), and disease. Based on the investigation and interviews, it was found that the villages had a high incidence rate (23%) of liver cancer, esophageal cancers, lung cancer, and other cancers in residents. The mean body weight of the participants was 59.8 kg, though they ranged from 46 to 69 kg (Table S1). All the participants were local farmers and provided written consent.

### 2.2. Sampling, Pre-Treatment and Analysis

All participants provided some of samples of home-grown rice, and vegetables. Taking into account regional consumption practices, the food crops included 20 home-grown rice for major grains and 38 vegetables. Vegetables such as Hon Tsai Tai broccoli (*Brassica compestris* L. var. purpurea Bailey), Chinese cabbage peduncle (*Brassica campestris* L.), asparagus lettuce (*Lactvca saiva* L.), scallion (*Allium fistulosum* L.), edible amaranth (*Amaranthus mangostanus* L.), Chinese cabbage (*Brassica Pekinensis* L.), lettuce (*Lactuca sativa* var ramosa), pak choi (*Brassica Chinensis* L.), spinach beet (*Beta vulgari* L.), spinach (*Spinacia oleracea* L.), carrot (*Daucus carota* L.), and crown daisy (*Chrysanthemum soronarium* L.) were provided or collected from randomly selected homes in three villages. At each sampling site, three to five of the same vegetable sub-samples were combined into a composite sample. Moreover, 16 market rices, purchased from the local farmer’s market, were used as a control group. In addition to food crops, 18 farmland soils, 4 river water, and 10 drinking water were collected from three villages around the impacted mine. Surface soils (depths of 0–20 cm) were sampled from farmlands. River water samples were collected from the Liuyang River. Drinking water was sampled from the well in randomly-selected homes and the shared wells in the village.

Edible parts of vegetables were washed thoroughly with Mili-Q water, and the fresh weights (fw) of the samples were recorded. All samples were dried, weighed again and recorded, then pulverized and stored in polythene zip-bags. The soil samples were air-dried at room temperature, then ground and sieved through a 1 mm stainless-steel mesh. The morning urine samples were collected from 30 participants and stored in 20 mL polyethylene bottles under low temperature during transport to the laboratory. In the laboratory, each urine sample was added to 2 mL concentrated HNO_3_. The urine samples were stored in a refrigerator with a monitored temperature of −20 °C until analysis.

Samples were microwave-digested (Anton-Paar PE Multiwave 3000) in a solution of 5 mL of HNO_3_ (69.5%, Merck, Kenilworth, NJ, USA) and 1 mL of H_2_O_2_ (30% *v*/*v*, Merck). After digestion, the solution was filtered with a 0.45-μm Teflon filter, and completed to 50 ml by ultra-pure distilled water. Inductively coupled plasma–mass spectrometry (ICP–MS, Agilent 7700x, Agilent Scientific Technology Ltd., Santa Clara, CA, USA) was used for analysis. The internal standards calibration was performed (72Ge and 115In). The limits of quantification (LOQ) of the proposed method were 0.03 (Cd) μg L^−1^, 0.16 (Pb) μg L^−1^ and 2.3 (As) μg L^−1^. For statistical analysis, if the concentration was below the LOQ it was set to 50% of the detection limit.

### 2.3. Evaluation of Bioaccessibility

Metal(loid) bioaccessibility assessment was performed using the physiologically based extraction test (PBET) method described by Intawongse and Dean [20]. Briefly, it mimics human digestion, including two simulated digestive processes in the stomach and small intestines. It was carried out using 0.5 g of rice or vegetable (pak choi) samples in a 100 mL screw-cap Sarstedt tube in which 50 mL of freshly prepared gastric solution was added. Rice and pak choi samples included three individual replications. Firstly, the gastric solution contained 1.25 g L^−1^ pepsin, 0.50 g L^−1^ maleic acid, 0.50 g L^−1^ citric acid, 420 μl L^−1^ DL-lactic acid and 500 μl L^−1^ acetic acid dissolved in water, and the pH was adjusted to 1.5 with HCl. Secondly, in the gastrointestinal stage, the amounts of 15 mg pancreatin and 52.5 mg bile salts were added in the sample tube and the pH of the mixture was adjusted to pH 7 with saturated NaHCO_3_. All of the samples were incubated at 37 °C with orbital-horizontal shaking and centrifuged to obtain supernatant before analysis.

### 2.4. Quality Assurance and Control

Glassware was properly cleaned, and the reagents were of analytical grade. Reagents blank determinations were carried out for each set of analysis to correct the instrument readings. Standard reference materials (SRM) obtained from the National Research Center for Certified Reference Materials (CRMs) of Beijing, China, including soil (GBW08303), spinach leaves (GBW10015), and rice powder (GBW10045) were used. Blank and drift standards were run after twenty determinations to calibrate the instrument. The recoveries related to the certified concentrations of the SRM for Cd, Pb, and As were 91.2%, 103%, and 98.4%, respectively.

### 2.5. Data Analysis

The bioaccessibility (%) of Cd, Pb and As were determined using the following equation [21]:

$$\mathrm{Bioaccessibility}\;\left(\%\right)=\frac{\mathrm{Bioaccesible}\;\mathrm{metal}\left(\mathrm{loid}\right)\;\mathrm{concentration}}{\mathrm{Total}\;\mathrm{metal}\left(\mathrm{loid}\right)\;\mathrm{concentration}}\times 100$$

In order to evaluate a short- or long-term potential hazardous exposure to Cd, Pb and As through consumption of food crops and water by consumers, the established daily intake (EDI) values for Cd, Pb and As based on the bioaccessibility data were evaluated using the following formula [2]:

$$\mathrm{EDI}=\frac{\mathrm{daily}\;\mathrm{consumption}\;\times \;\mathrm{bioaccessible}\;\mathrm{concentrations}\;\mathrm{of}\;\mathrm{metal}\left(\mathrm{loid}\right)s}{\mathrm{Body}\;\mathrm{weight}}$$

where rice and vegetable consumption results of 372 g day^−1^ and 274 g day^−1^, respectively, for local adults were obtained based on the survey.

All statistical analyses were performed using SPSS (Release 19.0, IBM, Chicago, IL, USA). All data were reported as the mean values, medians, or mean with standard deviation (SD) from several tested samples. Differences in metal(loid)s concentration between home-grown and market rice were determined using variance analysis based on Tukey’s multiple comparison using SPSS (20.0 package).

## 3. Result and Discussion

### 3.1. Metal(loid) Concentrations in Environmental Samples and Food Crops 

**Figure 2:**
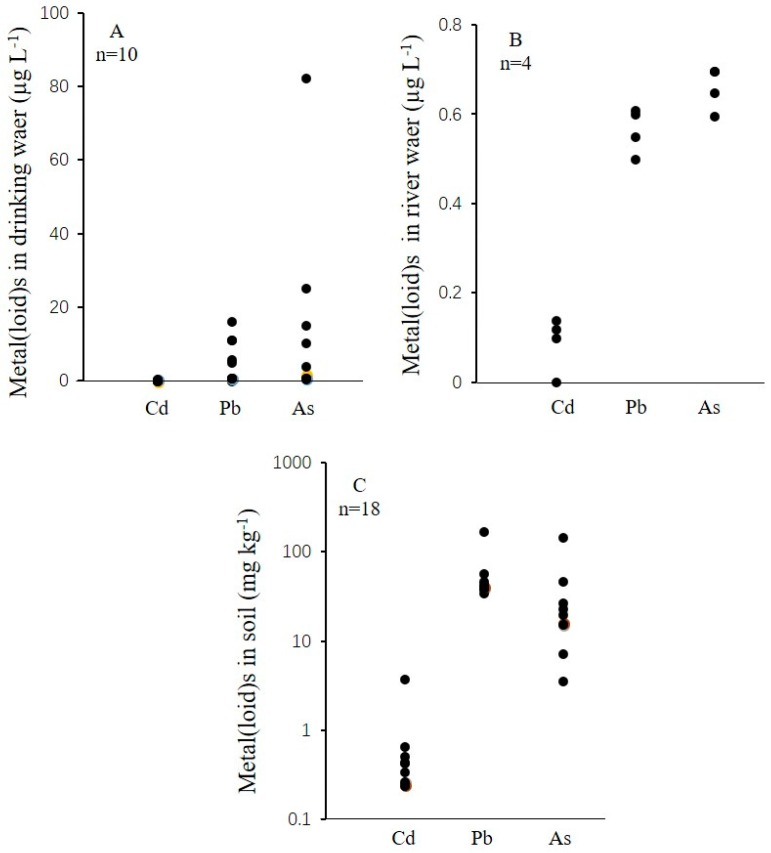
Cd, Pb and As concentrations in drinking water (**A**), river water (**B**) and soil samples (**C**)**.**

The characteristics and concentrations of Cd, Pb and As in 18 soils, ten drinking water, and four river water samples collected from the cohort study area are presented in Figure 2. The concentrations of Cd, Pb and As in river water (*n* = 4) were 0.001–0.14, 0.50–0.64 and 0.61–0.74 µg L^−1^, respectively, which were all below the Chinese threshold limit of irrigation water. For drinking water, the average concentrations of Cd, Pb and As were 0.05, 5.3 and 13 µg L^−1^, respectively. From the present results, the Cd and Pb concentrations in drinking water were below the safety limits. However, the As level in drinking water here exceeded the Chinese threshold limit (10 ug L^−1^) for drinking water. The highest levels of metal(loid)s in drinking water were generally collected from the farm homes near the mine. The Cd, Pb and As concentrations in farmland soils were 0.24–3.83, 35.1–173.7 and 23.7–148.7 mg kg^−^^1^, respectively (Figure 2). The average soil Cd concentration (0.72 mg kg^-1^) here exceeded the standard values (0.3 mg kg^−^^1^) for farmland of edible agricultural products of China [22]. The Pb concentrations for 11% of the soil samples were higher than the Chinese standard (80 mg kg^−^^1^) for farmland soils [22]. The average concentration of As (32.6 mg kg^−1^) also exceeded the Chinese limit (30 mg kg^−1^). The present results here indicated that soil Cd, Pb and As contamination due to the mining operation have become potential health threats in this area.

**Figure 3:**
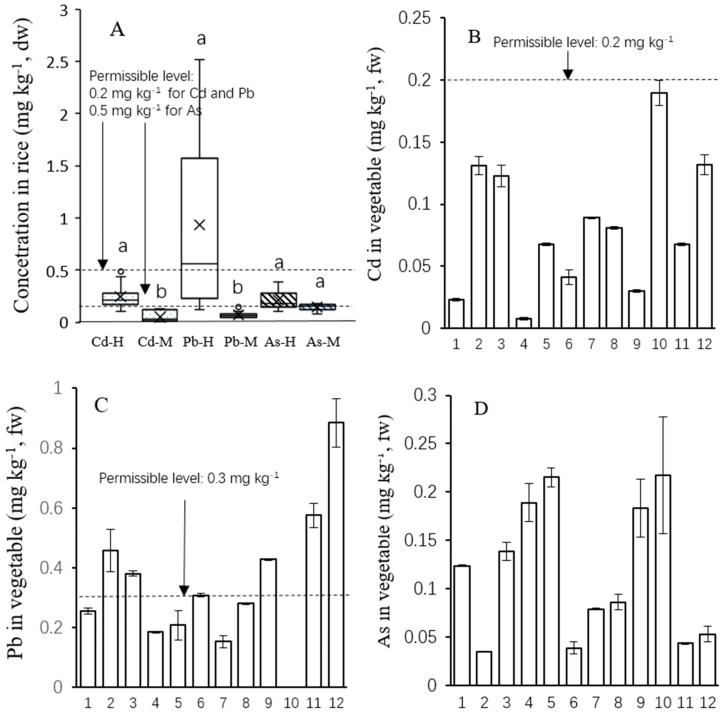
Cadmium, Pb and As concentrations in homegrown rice (H, *n* = 20) and market rice (M, *n* = 16) samples collected from study site (**A**), Cd, Pb and As concentrations in vegetable samples (**B**–**D**), respectivelyl. The vegetables included 1. Hon Tsai Tai (*n* = 4); 2. Chinese cabbage peduncle (*n* = 3); 3. Asparagus lettuce (*n* = 4); 4. Scallion (*n* = 3); 5. Edible amaranth (*n* = 3); 6. Chinese cabbage (*n* = 3); 7. Lettuce (*n* = 3); 8. Pak choi (*n* = 4); 9. Spinach beet (*n* = 3); 10. Spinach (*n* = 2); 11. Carrot (*n* = 3); 12. Crown Daisy (*n* = 3). Boxes represent the 25–75th percentiles. The error bars indicate the standard deviation. Different letters on boxes in (**A**) indicate significant differences (*p* < 0.05) between means of home-grown and market-purchased rice.

The metal(loid) concentration in home-grown and market rices are presented in Figure 3A. Significantly higher concentrations of Cd (*p* < 0.05) were observed in home-grown rice (0.11–0.43 mg kg^−1^) compared to market rice (0.01–0.13 mg kg^−1^). Among local-grown rice (*n* = 20), 68% exceeded the Chinese Cd maximum permittable value of 0.20 mg kg^−1^, whereas all market rice was below the limit. Elevated Pb concentrations were also observed in local rice (0.14–2.51 mg kg^−1^), with 49% of the market-purchased rice samples exceeding the Chinese Pb threshold value of 0.20 mg kg^−1^. The results of this study suggested that local-grown rice could accumulate high concentrations of Pb. Although the total Pb concentration was relatively low in the soil, the Pb concentrations in some vegetables exceeded the food safety limits. Similarly, high concentrations of Cd (0.02–0.61 mg kg^−1^) and Pb (0.03–2.11 mg kg^−1^) have been found in rice produced from some sites impacted by mining and smelting activities in China [2,20]. The concentrations of As in local rice (0.13–0.24 mg kg^−1^ dw) and market purchased rice (0.07–0.18 mg kg^−1^ dw) were analyzed, averaging 0.21 and 0.15 mg kg^−1^ dw, respectively, which were lower than the Chinese As threshold value (0.50 mg kg^−1^, dw). 

For vegetables, Cd concentrations (0.008–0.19 mg kg^−1^ fw) were analyzed, with an average of 0.08 mg kg^−1^ fw (Figure 3B), which were below the Chinese threshold value (0.2 mg kg^−1^, fw) for leafy vegetables. Among different species, spinach (0.19 mg kg^−1^, *n* = 2) and pakchoi (0.14 mg kg^−1^, *n* = 4) contained high Cd concentrations. These values are similar to those reported by Zhuang et al. [9] that leafy vegetables may contain elevated Cd compared to other vegetables. The concentrations of Pb in vegetable (0.001–0.88 mg kg^−1^ fw) were analyzed, with an average of 0.36 ± 0.15 mg kg^−1^ fw (Figure 3C). The highest value was found in crown daisy and the lowest in spinach. The present values in 42% vegetable samples were more than three times higher than the Chinese Pb limit of 0.3 mg kg^−1^ fw for leafy vegetables. In this study, the As concentrations in vegetables varied from 0.035–0.22 mg kg^−1^ fw (Figure 3D), which were all below the Chinese As limit of 0.50 mg kg^−1^ fw.

### 3.2. Metal(loid)s Bioaccessibility in Rice and Vegetables

**Figure 4:**
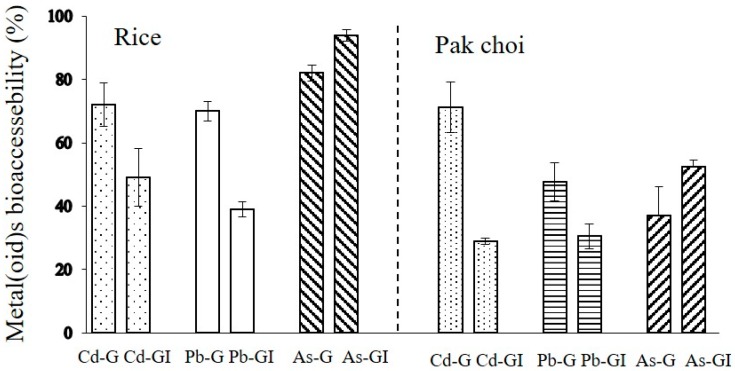
The metal(loid)s bioaccessibility (%, *n* = 3, means ± SD) in rice and pak choi established by the physiologically based extraction test (PBET) method (G is for gastric phase and I is for gastrointestinal phase).

Human exposure to metal(loid)s is influenced by both metal(loid) bioaccessibility or bioavailability and total concentration in the foods [23,24]. The oral bioaccessibility of Cd, Pb and As measured in the gastric and gastrointestinal fractions for rice and vegetables defined by PBET methods are presented in Figure 4.

In rice, the average Cd, Pb and As bioaccessibilities in the gastric phase were 72%, 70% and 82%, respectively. In the gastrointestinal phase, the average Cd, Pb and As bioaccessibilities were 49%, 39% and 94%, respectively. These values are similar to our previous reports by Zhuang et al. [23]. With respect to pak choi, the average bioaccessibility values for Cd, Pb and As were 71%, 48% and 37% in the gastric fraction, whereas the bioaccessibility values for the gastrointestinal fraction were 29%, 30.5% and 52.5%, respectively. These present results were higher or similar to metal(loid) bioaccessibility from vegetables reported by Hu et al. [25] and Fu and Cui [26]. Compared with the values in several types of raw vegetables by Zhuang et al. [17], the average bioaccessibility for the gastric and gastrointestinal fractions were similar, in which Pb bioaccessibility varied from 10 to 60% in the gastric phase and from 13 to 39% in the gastrointestinal phase. So, high contamination level in rice or other food crops could explain a large proportion of variation in the predicted bioaccessibility levels in our previous study [23]. In contrast to Cd and Pb, As bioaccessibility in the gastrointestinal fraction was higher than those during the gastric fraction. It is likely that in the simulated intestinal juices, some enzymes from the pancreas and bile are involved in the breakdown of poly-saccharides into monosaccharide, and free amino acids and small peptides with a chain length of two to six amino acid residues resulting from the cleavage of denaturalized proteins [27]. The As percentage in the gastrointestinal phase is remarkably higher than bioaccessibility of both Cd and Pb. These results further suggested that the gastrointestinal fraction of As played an important role in the solubilization during the digestion process. 

### 3.3. Concentration of Cd, Pb and As in the Urine Samples

**Figure 5:**
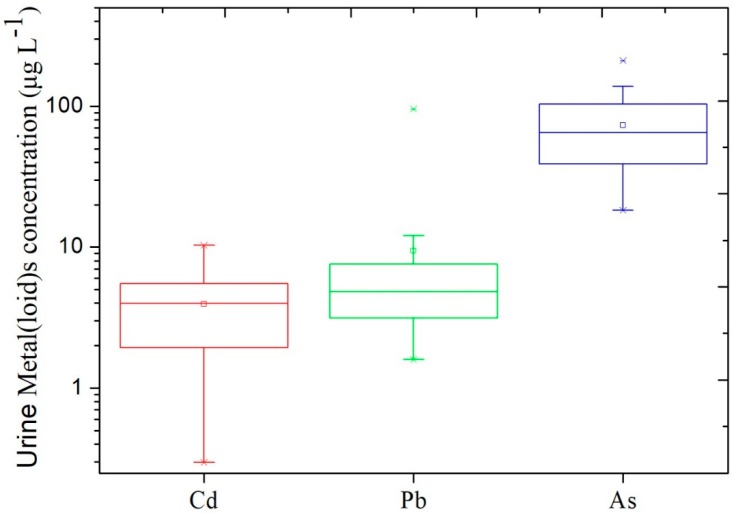
Metal(loid) concentrations in urine samples collected from the local residents (*n* = 30).

The median concentration of Cd in urine was 3.99 µg L^−1^ (ranging from 0.3 to 10.3 µg L^−1^) (Figure 5). The data we reported here were higher than those reported in the United States (median: 0.32–0.40 µg L^−1^ in adults) [28], in China (median: 0.2–0.64 µg L^−1^ in adults) [14] and in South Korea (median: 0.66 µg L^−1^ for male, 0.73 µg L^−1^ for female) [29]. Urinary Pb concentrations of the participants ranged from 1.59 to 18.9 µg L^−1^ (except for an extreme value: 95.77 µg L^−1^), which were similar to or slight higher than those from other cities of China (2.75–7.52 µg L^−1^) [30]. The concentration of total As in urine ranged from 18.3 to 211.3 µg L^−1^ (Figure 5), lower than the values 78 to 459 µg L^−1^ in Northern Chile reported by Diaz et al. [31]. These comparisons indicated that the local residents in our study were under significantly higher environmental Cd exposure. Zhao et al. [24] also suggested that incorporating Cd bioavailability in foods to evaluate dietary intake of Cd is a valuable tool to accurately assess Cd exposure and associated human health risk. Comparing the metal(loid)s in urine samples from each individual, we found that urine metal(loid)s levels was not associated with age of the local residents. This was different from the results by Sun et al. [14]. It is likely that there were not enough urine samples involved in the present study. Moreover, lack of information about U-creatinine was a limitation of this study. Thus, further research is needed to integrate U-creatinine analysis with sufficient urine samples collected from the contaminated sites.

### 3.4. Contribution of Food Samples to Metal(loid)s Exposure in Humans

Since dietary intake is considered as one of the main pathways of metal(loid) exposure to humans, the influence of food sources to aggregate metal(loid) exposure, food metal(loid) intake was calculated based on average total concentration, average metal(loid)s bioaccessibility, and consumption of rice, vegetables and drinking water (Table 1). Based on the field survey and the consolidated bioaccessibility data, when considering several main dietary routes, overall Cd exposure was 1.14 μg kg^−1^ bw day^−1^, higher than the JECFA (Joint FAO/WHO Expert Committee on Food Additives) threshold of 0.83 μg kg^−1^ bw day^−1^. The EDI value of Pb exposure from rice, vegetables and drinking water was 3.96 μg kg^−1^ day^−1^, exceeding the EFSA (European Food Safety Authority) threshold (1.5 μg kg^−1^ bw day^−1^) by 2.6 times. The overall As exposure was 1.88 μg kg^−1^ bw day^−1^, below the JECFA provisional tolerable daily intakes (3 μg kg^−1^ bw day^−1^). Due to the high total concentration, rice represented the largest source of Pb exposure, accounting for 210%, followed by vegetable (40.6%), with little contribution rate from drinking water (13%). Similarly, large contribution rates of Cd intake from homegrown rice and vegetables were 114% and 22.2%, respectively. These values were lower than the exposure contribution of Cd (212%) by rice purchased from local markets around a mining area in our previous study [23]. Based on the above statistical data, If the intake of metal(loid)s through locally produced rice and vegetables is taken into account, the great potential health threat cannoned be overstated: The effect of this type of consumption should not be overlooked.

In China, metal(loid) contaminated rice and vegetables produced in the polluted soils around mining-impacted areas are sold at the markets all over the country, resulting in great human health risk for the consumers, which are not restricted to the polluted area [2,26]. Given the situation of contaminated rice and vegetables, the health risk of Pb and Cd poisoning is the greatest for people who eat rice and vegetables several times a day, however eating less rice or vegetables is not an option in many parts of the world, where it is an irreplaceable part of culture, diet and lifestyle. In the future studies, a strategy should be developed to modulate dietary metal(loid) exposure via decreasing bioaccessibility or bioavailability in food crops.

## 4. Conclusions

The present study showed that rice Cd and Pb, vegetable Pb and soil Cd levels exceeded the maximum permissible values. Arsenic levels in several types of environmental samples and urine samples were below the permissible limit. Cadmium, Pb and As bioaccessibilities in rice were 72%, 70%, and 82% in gastric phases, respectively. Cadmium, Pb and As bioaccessibilities in rice were 49%, 39%, 94% in gastrointestinal phases, respectively. Vegetable samples showed relatively lower metal(loid) bioaccessibility than those in rice. The median concentration of Cd in urine was ten times higher than that reported in the literature. Based on the bioaccessibility data, rice represented the largest source of Cd and Pb exposure, followed by vegetables. It suggested that the local residents were under significantly high environmental Cd or Pb exposure and its health risk cannot be overlooked.

## Figures and Tables

**Table 1:** Calculated values of established diary intake (EDI) and contribution rate (%) for Cd, Pb and As based on the gastrointestinal metal(loid)s bioaccessibility data via consumption of rice, vegetables, drinking water for an adult with body weight of 60 kg.

Metal(loid)	Parameter	Rice	Vegetables	Water	Over Exposure
Cd	Total (mg kg^−1^)	0.254	0.081 (fw)	0.0003 mg L^−1^	
	Bioaccessibility (%) ^a^	60.5	50	100	
	Intake rate (day^−1^) ^b^	372 g	274 g	2 L	
	EDI (µg kg^−1^ bw day^−1^)	0.95	0.18	0.01	1.14
	Contribution rate (%) ^c^	114	22.2	1.2	
Pb	Total (mg kg^−1^)	0.93	0.34 (fw)	0.0059	
	Bioaccessibility (%)	54.5	39	100	
	Intake rate (day^−1^)	372 g	274 g	2 L	
	EDI (µg kg^−1^ bw day^−1^)	3.15	0.61	0.20	3.96
	Contribution rate (%) ^d^	210	40.6	13.1	
As	Total (mg kg^−1^)	0.204	0.164 (fw)	0.0129	
	Bioaccessibility (%)	88	44.8	100	
	Intake rate (day^−1^)	372 g	274 g	2 L	
	EDI (µg kg^−1^ bw day^−1^)	1.11	0.34	0.43	1.88
	Contribution rate (%) ^e^	37.1	11.1	14.3	

^a^ The average bioaccessibility of gastric and gastrointestinal phase for the rice and pakchoi and the reports from Zhuang et al. [17]. ^b^ Daily intakes for rice and vegetable are based on the survey from this cohort study. ^c^ For Cd, the provisional tolerable monthly intake (PTMI) 0.025 mg kg^−1^ bw on a monthly basis according to JECFA; ^d^ For Pb, the provisional tolerable intake (PTDI) of 1.5 µg kg^−1^ bw day^−1^ according to European Food Safety Authority (EFSA); ^e^ The provisional tolerable weekly intake (PTWI) of 21 µg kg^−1^ bw (equivalent to 3 µg kg^−1^ bw day^−1^) for As according to JECFA. When calculating metal(loid)s intake via water ingestion, the average metal(loid)s bioaccessibility in drinking water was assumed at 100%.

## Supplementary Materials

The following are available online at http://www.mdpi.com/1660-4601/15/8/1573/s1, Table S1: The results from the field survey.
