# Supplementary material for: Oral Bioaccessibility and Exposure Risk of Metal(loid)s in Local Residents Near a Mining-Impacted Area, Hunan, China
Source: Int J Environ Res Public Health. 2018 Jul 25;15(8):1573. doi: 10.3390/ijerph15081573 (PMC6121664; doi:10.3390/ijerph15081573)
Supplement: Supplementary file 1 [file ijerph-15-01573-s001.pdf]

Oral Bioaccessibility and Exposure Risk of Metal(loid)s in Local Residents Near a Mining-Impacted Area, Hunan, China

Ping Zhuang [1], Shuo Sun [1,2], Yingwen Li [1], Feng Li [1,2], Bi Zou [1], Yongxing Li [1], Hui Mo [1] and Zhian Li [1,*]

Table S1. The results from the field survey.

Participants	Age	Gender	Weight (kg)	Rice (g/d)	Vegetable (g/d)	Urine (ml)
1	60	Male	58.3	380	300	10
2	24	Female	51.6	340	280	10
3	46	Male	69.4	440	270	10
4	61	Male	62.3	375	280	10
5	48	male	65	395	295	10
6	28	Female	56.2	345	275	10
7	62	Male	59.3	358	260	10
8	60	Female	53.1	340	270	10
9	47	Female	55.7	340	260	10
10	26	Female	49.4	340	280	10
11	80	Male	63.7	350	280	10
12	40	Male	66.8	420	270	10
13	48	Female	46.4	330	260	10
14	50	Male	63.5	360	260	10
15	58	Male	68.6	450	295	10
16	64	Female	49.8	335	290	10
17	64	Male	58.9	380	270	10
18	42	Female	51.7	355	280	10
19	31	Male	65.1	350	260	10
20	68	Male	68.3	420	255	10
21	48	Male	59.8	375	280	10
22	66	Male	69.4	385	270	10
23	67	Male	67.5	380	275	10
24	45	Female	52.9	350	290	10
25	80	Female	47.9	330	280	10
26	55	Male	68.7	450	295	10
27	42	Male	63.9	385	280	10
28	46	Female	57.8	345	245	10
29	59	Male	67.4	395	280	10
30	48	Female	56.2	355	245	10
